# Severe Post‐Tuberculosis Bronchiectasis With Bullous Lung Disease Complicated by Acute Infective Exacerbation: A Case Report From Uganda

**DOI:** 10.1002/ccr3.72854

**Published:** 2026-06-02

**Authors:** Abdisalam Ahmed Sandeyl, Farah Dubad Abdi, David Elia Saria, Grace Akumu Oling, Abdisamad Guled Hersi, Hailemariam Kassahun Bekele, Abdalla Ahmed Deifa

**Affiliations:** ^1^ Internal Medicine Department Kampala International University, Western Campus Ishaka‐Bushenyi Uganda

**Keywords:** bronchiectasis, chest computed tomography, infective exacerbation, post‐tuberculosis lung disease, structural lung disease, Uganda

## Abstract

We report a case of severe post‐tuberculosis lung disease (PTLD) in a 60‐year‐old Ugandan man with a six‐year history of suboptimally treated pulmonary tuberculosis (PTB), who presented with a three‐week history of productive cough, yellow foul‐smelling sputum occasionally streaked with blood, dyspnea, pleuritic chest pain, fatigue and progressive exercise intolerance. Imaging revealed extensive structural lung damage characterized by bronchiectasis, multiple bullae, and fibrotic changes, more pronounced in the left lung. Laboratory evaluation showed leukocytosis with neutrophilic predominance, and sputum culture grew *Pseudomonas aeruginosa* and *Streptococcus pneumoniae* . A diagnosis of post‐tuberculosis structural lung disease complicated by acute infective exacerbation of bronchiectasis was made. Treatment with intravenous empirical antibiotics followed by culture‐directed therapy, along with supportive care, led to clinical improvement and reduction in respiratory symptoms. This case highlights the severe structural sequelae of suboptimally treated PTB and the importance of early recognition and appropriate management of infective exacerbations, particularly in resource‐limited settings.

## Key Clinical Message

Post‐tuberculosis lung disease may present with severe structural lung damage and acute infective exacerbations of bronchiectasis resulting in significant respiratory morbidity. Early recognition, chest CT evaluation, and prompt culture‐directed antibiotic therapy are essential to reduce morbidity, particularly in resource‐limited settings.

**Abbreviations**
COPD: chronic obstructive pulmonary disease
CT: computed tomography
PTB: pulmonary tuberculosis
PTLD: post‐tuberculosis lung disease

## 1 Introduction

Post‐tuberculosis lung disease (PTLD) is an increasingly recognized cause of chronic respiratory morbidity, particularly in regions with a high burden of pulmonary tuberculosis (PTB) [1]. It encompasses a broad spectrum of structural abnormalities, including airway distortion, fibrosis, and parenchymal destruction, which may persist despite microbiological cure. Among these, bronchiectasis is one of the most common and clinically significant sequelae, predisposing patients to recurrent infections and progressive lung damage [2].

Clinically, PTLD often presents with chronic cough, sputum production, dyspnea, and hemoptysis, frequently mimicking active PTB or other chronic respiratory conditions [3]. Imaging plays a central role in evaluation, with chest radiography and computed tomography typically demonstrating bronchiectasis and fibrotic changes, with features of advanced disease such as architectural distortion [4]. These structural abnormalities create a favorable environment for persistent microbial colonization and recurrent infections with common respiratory pathogens, resulting in acute infective exacerbations [5].

In resource‐limited settings, delayed diagnosis and suboptimal treatment of PTB remain common and contribute substantially to the burden of PTLD. Diagnostic challenges are further compounded by overlapping clinical features with active infection and limited access to advanced imaging and microbiological investigations [6].

We report a case of severe PTLD complicated by an acute infective exacerbation of bronchiectasis in a Ugandan patient, emphasizing the importance of early recognition and comprehensive management of long‐term pulmonary sequelae arising from suboptimally treated PTB in resource‐limited settings.

## 2 Case Presentation

### 2.1 Case History and Examination

A 60‐year‐old man with a six‐year history of suboptimally treated PTB presented to Kampala International University Teaching Hospital with a three‐week history of progressively worsening productive cough associated with copious yellow, foul‐smelling sputum occasionally streaked with blood. He reported premature discontinuation of anti‐tuberculosis therapy after 3 months despite a prescribed six‐month treatment course. The patient also reported increasing shortness of breath, mild pleuritic chest pain, progressive fatigue, and reduced exercise tolerance, which limited his ability to perform routine daily activities. He denied significant hemoptysis, night sweats, weight loss, or recent contact with individuals with active PTB. He reported previous similar but milder episodes that responded to antibiotic therapy. He was a former smoker with a significant history of tobacco use, having quit 2 years prior to presentation. His HIV status was confirmed negative.

On examination, the patient was in severe respiratory distress and required supplemental oxygen. Vital signs revealed blood pressure of 110/70 mmHg, pulse rate of 97 beats per minute, and oxygen saturation (SpO_2_) of 76% on room air. The patient was hypoxic with features of impending respiratory failure, necessitating urgent supportive management.

Respiratory examination demonstrated reduced chest expansion predominantly over the left upper lung zone, with increased tactile vocal fremitus on the left side. Percussion revealed relative dullness over the left upper lung zone. Auscultation revealed reduced air entry over the left upper lung zone with diffuse wheezing, and a mixture of fine and coarse crackles over the left lung and right lower lung zone. Grade 3 digital clubbing with increased nail curvature (parrot‐beak appearance) was observed. Use of accessory muscles of respiration was noted. Other systemic examinations were unremarkable.

### 2.2 Investigations and Differential Diagnosis

Laboratory evaluation revealed leukocytosis with a white blood cell count of (13.6 × 10^9^/L; reference range 4–11 × 10^9^/L) with granulocyte predominance of (88%; reference range 40%–75%), consistent with an acute bacterial infection. Renal and liver function tests, serum electrolytes, and urinalysis were within normal limits. Sputum testing using GeneXpert for *Mycobacterium tuberculosis* was negative. These findings reduced the likelihood of active PTB and supported a diagnosis of post‐tuberculosis sequelae. Sputum culture grew *Pseudomonas aeruginosa* and *Streptococcus pneumoniae* . Both organisms demonstrated sensitivity to piperacillin‐tazobactam and levofloxacin.

**FIGURE 1:**
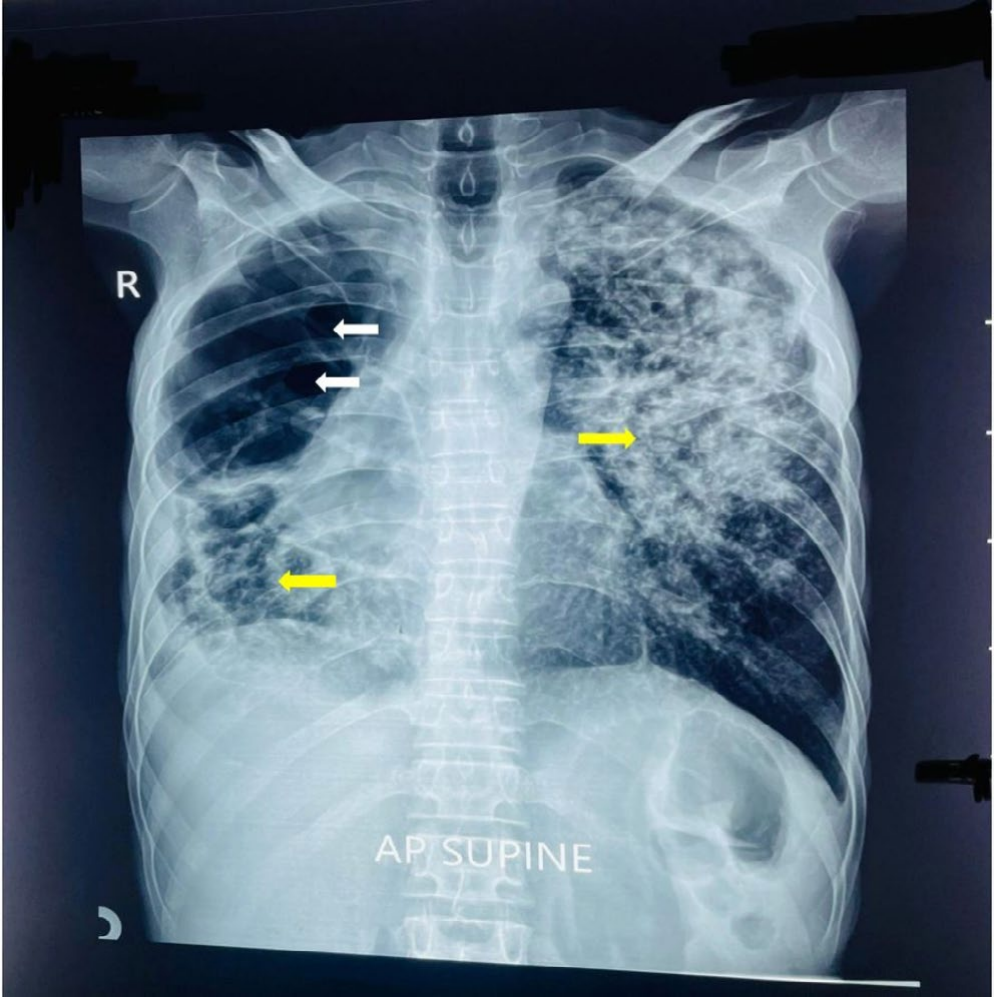
*Chest radiograph* (*anteroposterior view*) *demonstrating extensive bilateral structural lung disease*. Heterogeneous opacities are seen predominantly in the left upper lung zone, with additional patchy opacities in the right mid and lower zones (yellow arrows). Multiple rounded lucent areas in the right upper lung zone suggestive of bullous changes (white arrows).

Chest radiography demonstrated extensive heterogeneous opacities predominantly involving the left upper lobe, with multiple lucent areas consistent with bullous changes and associated architectural distortion. Additional patchy opacities were noted in the right middle and lower lung zones, suggestive of superimposed inflammatory or infective changes. No clear air–fluid levels were identified (Figure 1).

**FIGURE 2:**
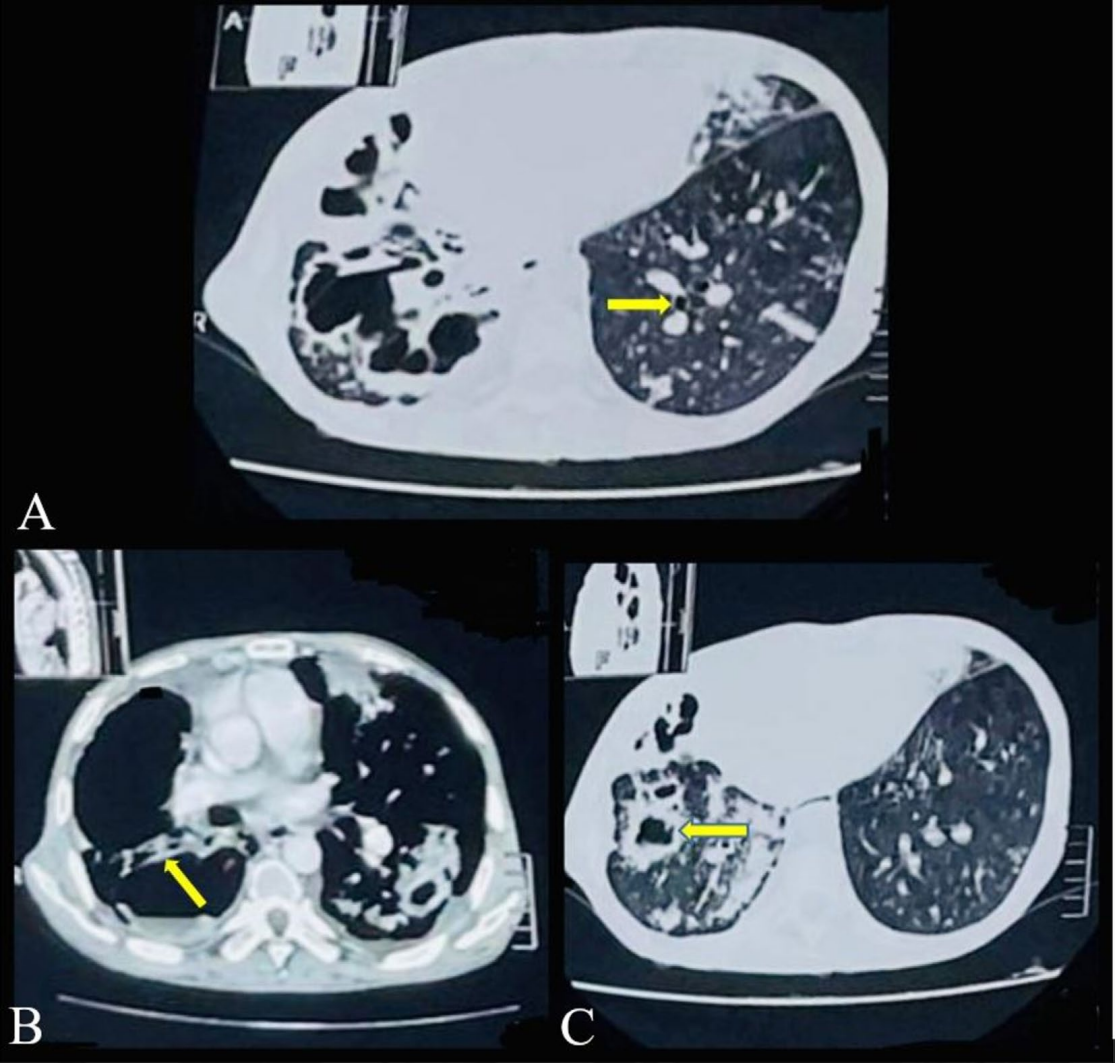
*Axial chest CT images demonstrating extensive bilateral structural lung disease consistent with post‐tuberculosis sequelae*. (A) Cystic bronchiectasis with clustered dilated airways showing a signet ring appearance. (B) Tram‐track appearance of bronchiectasis with thickened dilated bronchi. (C) Large bullae, more prominent in the right lung.

Computed tomography (CT) of the chest revealed extensive bilateral but asymmetrical lung involvement characterized by cystic bronchiectasis with signet ring appearance, tram‐track opacities, a large bullae more prominent in the right lung, and associated fibrotic changes. These findings were more pronounced in the left lung and were accompanied by architectural distortion, consistent with chronic destructive lung disease (Figure 2A–C).

Based on the clinical history and imaging findings, a diagnosis of post‐tuberculosis structural lung disease complicated by an acute infective exacerbation of bronchiectasis was made.

Differential diagnoses considered included active or reactivation PTB, Chronic obstructive pulmonary disease (COPD) with infective exacerbation, Non‐tuberculous mycobacterial infection, and Lung abscess. Active PTB was considered given the prior history; however, it was less likely due to negative GeneXpert testing, absence of systemic symptoms, and imaging findings consistent with chronic structural changes rather than active disease. COPD was strongly considered given the significant smoking history and overlapping respiratory symptoms; however, the extent and distribution of bronchiectasis, associated fibrotic changes, and marked architectural distortion were disproportionate to typical COPD findings. The presence of digital clubbing, which is not a typical feature of COPD, further supported an alternative diagnosis. Non‐tuberculous mycobacterial infection was considered in view of underlying structural lung disease but was less likely in the absence of supportive microbiological evidence and lack of characteristic imaging features such as nodular or tree‐in‐bud opacities. Lung abscess was considered due to foul‐smelling sputum but was unlikely in the absence of a localized cavitary lesion with an air–fluid level. Overall, the findings favored a diagnosis of post‐tuberculosis structural lung disease with superimposed infective exacerbation of bronchiectasis.

### 2.3 Treatment and Outcome

The patient was initially started on empirical intravenous ceftriaxone (1 g twice daily) and metronidazole (500 mg three times daily) to provide broad‐spectrum antimicrobial coverage, including anaerobic organisms. Following sputum culture and sensitivity results, antibiotic therapy was escalated to intravenous piperacillin‐tazobactam (4.5 g four times daily) and levofloxacin (500 mg twice daily), targeting *Pseudomonas aeruginosa* and *Streptococcus pneumoniae* . Supportive management included supplemental oxygen therapy, regular chest physiotherapy to facilitate airway clearance, and nebulized bronchodilator therapy with Salbutamol and Ipratropium bromide (5 mg/2.5 mg four times daily).

Over the course of 1 week, the patient demonstrated clinical improvement, with reduced sputum production, decreased dyspnea, improved oxygenation, and reduced respiratory distress on examination. The patient also reported improved exercise tolerance and ability to perform routine daily activities. He was discharged on oral levofloxacin (750 mg once daily for 1 week) and azithromycin (500 mg three times weekly for 3 months) as long‐term therapy for bronchiectasis. He was also advised to receive influenza and pneumococcal vaccinations, with outpatient follow‐up scheduled.

Due to limited availability of pulmonary function testing at our institution, the patient was referred to Mbarara Regional Referral Hospital for spirometry prior to follow‐up review. At the three‐week follow‐up visit, the patient reported sustained improvement in cough, dyspnea, and exercise tolerance, with no recurrent infective symptoms. Influenza and pneumococcal vaccinations were administered at this visit. Spirometry demonstrated a moderate obstructive ventilatory pattern (FEV1/FVC < 0.7, FEV1 70% predicted), consistent with moderate airflow limitation and further supporting chronic airway disease.

## 3 Discussion

### 3.1 Burden and Clinical Significance of Post‐Tuberculosis Lung Disease

The long‐term consequences of PTB extend beyond microbiological cure, with PTLD increasingly recognized as a major contributor to chronic respiratory morbidity [1]. Longitudinal studies show that many patients develop persistent respiratory symptoms, functional impairment, and radiological abnormalities despite completing standard anti‐tuberculosis therapy [7].

The clinical burden of PTLD is substantial. Affected individuals commonly experience chronic cough, dyspnea, and reduced exercise tolerance, which are associated with impaired quality of life and functional limitation. PTLD is also linked to increased healthcare utilization, including recurrent hospitalizations, and has been associated with higher long‐term mortality compared to the general population [8].

Despite this, PTLD remains under‐recognized in routine practice, particularly in high‐burden settings where emphasis is placed on diagnosis and treatment of active PTB with limited post‐treatment follow‐up [9].

### 3.2 Radiological Spectrum and Severe Structural Destruction

Cross‐sectional imaging with computed tomography (CT), particularly high‐resolution techniques, is the most sensitive approach for characterizing structural abnormalities in PTLD, offering superior detection compared to chest radiography [4]. Typical findings include airway dilatation, fibrotic bands, volume loss, and architectural distortion, reflecting chronic parenchymal injury. Among these, bronchiectasis is the most consistently reported abnormality and has been shown to correlate with both symptom burden and functional impairment [10].

With disease progression, fibrotic changes may lead to irreversible lung remodeling and architectural distortion, reflecting advanced parenchymal injury [4]. Emphysematous and bullous changes have also been described, although they are less commonly emphasized in post‐tuberculosis lung disease and are more often associated with severe or prolonged disease [11].

In this case, the combination of bronchiectasis, fibrotic remodeling, and multiple bullae represents advanced structural lung disease. Such extensive architectural distortion is associated with significant physiological consequences, including impaired gas exchange, ventilation–perfusion mismatch, and an increased risk of respiratory failure [12].

### 3.3 Microbiology and Pathogenesis of Infective Exacerbations

Chronic airway damage in PTLD creates a favorable environment for persistent microbial colonization and recurrent infective exacerbations. Structural abnormalities such as bronchial dilatation and impaired mucociliary clearance facilitate bacterial persistence, while ongoing inflammation contributes to altered local immune responses [5]. In non‐cystic fibrosis bronchiectasis, chronic bacterial colonization has been closely linked to sustained airway inflammation and progressive lung injury [13].

The microbiological profile of bronchiectasis is well characterized, with common pathogens including *Pseudomonas aeruginosa* , *Haemophilus influenzae* , and *Streptococcus pneumoniae* [14]. Among these, *Pseudomonas aeruginosa* is consistently associated with more severe disease, increased exacerbation frequency, and worse clinical outcomes. Its ability to form biofilms and evade host immune mechanisms enables persistent colonization and contributes to chronic infection [15].

The isolation of both *Pseudomonas aeruginosa* and *Streptococcus pneumoniae* in this case supports the polymicrobial nature of infections in structurally abnormal lungs and reflects a high burden of airway colonization.

### 3.4 Diagnostic Challenges and Differential Diagnosis

PTLD presents significant diagnostic challenges due to its clinical and radiological overlap with active or recurrent PTB, particularly in high‐burden settings [16]. Patients commonly present with chronic respiratory symptoms such as cough, sputum production, hemoptysis, and dyspnea, which are non‐specific and may mimic ongoing infection. This overlap may lead to misclassification as treatment failure or disease relapse, resulting in unnecessary retreatment or delayed recognition of chronic structural lung disease [3].

Other important differential diagnoses include non‐tuberculous mycobacterial infection and COPD, both of which may present with persistent respiratory symptoms and structural lung abnormalities. Non‐tuberculous mycobacterial disease may closely mimic PTLD both clinically and radiologically, particularly in patients with underlying bronchiectasis, while COPD may present with similar chronic respiratory symptoms in individuals with a history of smoking. Careful evaluation of imaging patterns, including the distribution of bronchiectasis and fibrosis, together with microbiological findings, is essential for accurate differentiation [4].

In Uganda and similar resource‐limited settings, these diagnostic challenges are further compounded by limited availability and high cost of chest CT imaging, restricted access to pulmonary function testing and advanced microbiological investigations, delayed healthcare‐seeking behavior, and inadequate long‐term post‐tuberculosis follow‐up. Consequently, diagnosis often relies heavily on clinical history, prior tuberculosis treatment status, basic imaging, and available sputum‐based investigations, resulting in many patients presenting with advanced structural lung disease and recurrent infective exacerbations [6].

### 3.5 Management Strategies and Long‐Term Care

Management of PTLD is largely extrapolated from established approaches to bronchiectasis and other chronic respiratory conditions, given the absence of disease‐specific guidelines [17]. Acute infective exacerbations require prompt initiation of empirical antibiotic therapy, followed by adjustment based on microbiological culture and sensitivity results, as delayed or inappropriate treatment has been associated with worse clinical outcomes [18].

Airway clearance strategies, including chest physiotherapy, play a central role in management by facilitating mucus clearance, reducing bacterial load, and improving respiratory symptoms [19]. In patients with associated airflow limitation, bronchodilator therapy may provide additional symptomatic benefit and improve functional status [20]. In the present case, culture‐directed antibiotic therapy combined with airway clearance and bronchodilator use resulted in clinical improvement.

Long‐term macrolide therapy has been shown to reduce exacerbation frequency through both antimicrobial and anti‐inflammatory effects and is recommended in selected patients with recurrent exacerbations [21]. Preventive measures, including vaccination against influenza and pneumococcal infection, are also important in reducing the risk of respiratory infections and disease progression [22].

### 3.6 Clinical Implications

This case underscores the long‐term consequences of suboptimal treatment of PTB and highlights the need for improved treatment adherence, structured post‐treatment follow‐up, and increased clinician awareness of PTLD.

Expanding the focus of PTB programs beyond microbiological cure to include long‐term respiratory outcomes is essential to reduce the burden of PTLD and improve quality of life.

## 4 Conclusion

We report a case of severe PTLD in a patient with a history of suboptimally treated PTB, presenting with an acute infective exacerbation of bronchiectasis and extensive structural lung destruction. This case highlights the potential for severe long‐term pulmonary sequelae following inadequate PTB treatment and underscores the importance of early recognition, culture‐directed therapy, and structured post‐treatment follow‐up in reducing disease burden, particularly in resource‐limited settings.

## Author Contributions

**Abdisalam Ahmed Sandeyl:** conceptualization, investigation, writing – original draft, writing – review and editing, project administration. **Abdisamad Guled Hersi:** investigation, resources. **David Elia Saria:** investigation, data curation. **Abdalla Ahmed Deifa:** supervision, writing – review and editing, resources. **Grace Akumu Oling:** investigation, writing – review and editing. **Hailemariam Kassahun Bekele:** investigation, writing – review and editing. **Farah Dubad Abdi:** conceptualization, writing – review and editing.

## Funding

The authors have nothing to report.

## Consent

Written informed consent was obtained from the patient for publication of this case report and any accompanying images.

## Conflicts of Interest

The authors declare no conflicts of interest.

## Data Availability

The data that support the findings of this study are available from the corresponding author upon reasonable request.
